# Genetic Diversity in *Echinococcus multilocularis* From the Plateau Vole and Plateau Pika in Jiuzhi County, Qinghai Province, China

**DOI:** 10.3389/fmicb.2018.02632

**Published:** 2018-11-05

**Authors:** Jian-qiu Li, Li Li, Yan-lei Fan, Bao-quan Fu, Xing-quan Zhu, Hong-bin Yan, Wan-zhong Jia

**Affiliations:** ^1^State Key Laboratory of Veterinary Etiological Biology, Key Laboratory of Veterinary Parasitology of Gansu Province, Key Laboratory of Veterinary Public Health of Agriculture Ministry, Lanzhou Veterinary Research Institute, Chinese Academy of Agricultural Sciences, Lanzhou, China; ^2^Jiangsu Co-innovation Center for Prevention and Control of Important Animal Infectious Disease, Yangzhou, China

**Keywords:** *E. multilocularis*, mitochondrial gene, genetic variation, haplotypes, plateau vole, plateau pika

## Abstract

The Qinghai-Tibet Plateau is a highly endemic area of alveolar echinococcosis where a series of intermediate hosts, especially voles and pikas, are infected with *Echinococcus multilocularis*. The metacestodes of *E. multilocularis* are fluid-filled, asexually proliferating cysts, and they are mainly found in the host's liver in the form of tumor-like growths. In this study, we investigated the genetic variations of *E. multilocularis* in four mitochondrial (mt) genes, namely, NADH dehydrogenase subunit 5 (*nad*5), adenosine triphosphate subunit 6 (*atp*6), cytochrome c oxidase subunit 1 (*cox*1), and NADH dehydrogenase subunit 1 (*nad*1). The complete *nad*5, *atp*6, *cox*1, and *nad*1 genes were amplified separately from each hydatid cyst isolate using polymerase chain reaction (PCR) and then sequenced. Phylogenetic trees were then generated based on the combined mt genes using MrBayes 3.1.2 and PAUP version 4.0b10. The results showed that thirty of 102 voles and two of 49 pikas were infected with *E. multilocularis*. The genetic variation distances among all *E. multilocularis* samples were 0.1–0.4%, 0.2–0.4%, 0.1–0.6%, and 0.1–0.4% for *nad*5, *atp*6, *nad*1, and *cox*1, respectively. Compared to previous studies of the genetic diversity of *E. multilocularis* based on the *cox*1 gene, the genetic distances within the same group were 1.3–1.7% (Mongolia strain), 0.6–0.8% (North American strain), 0.3–0.6% (European strain), and 0.1–0.4% (Asian strain). Based on concatenated sequences of the *nad*5, *atp*6, *cox*1, and *nad*1 genes all haplotypes were divided into two clusters. In conclusion, the genetic diversity of *E. multilocularis* based on mt genes on a small local area is at low level but between different regions with long distance and different ecological environment each other, the genetic diversity is at relatively high level; genetic variation is higher in the *nad*1 gene than that in the other three mt genes. The results on a local scale provide basic information for further study of the molecular epidemiology, genetic differences and control of *E. multilocularis* in Qinghai Province, China.

## Introduction

*Echinococcus multilocularis* is a small cestode that cause the parasitic zoonosis alveolar echinococcosis (AE), which was one of the 17 neglected tropical diseases (NTDs) prioritized by the World Health Organization (WHO) in 2012 (Agudelo Higuita et al., 2016). AE was discovered in the Nineteenth century; it has a considerable socioeconomic impact and until now has been sporadically found in humans (Nakao et al., 2007; Spahn et al., 2016). *E. multilocularis* is mainly distributed in holarctic regions, including North America (also found in southwestern Ontario), Europe, and Asia (Massolo et al., 2014; Oksanen et al., 2016; Deplazes et al., 2017; Trotz-Williams et al., 2017). Asia, especially China, has many highly endemic areas, such as Qinghai, Gansu, and Sichuan. *E. multilocularis* infects two kinds of hosts: the typical intermediate host (IH), a wide spectrum of mammalian species including small herbivorous, rodents (predominantly) and pikas, and the typical definitive hosts (DH), which are canids and mammalian species, including foxes, wolves, dogs, and cats (Vuitton et al., 2003; Deplazes et al., 2011; Hegglin and Deplazes, 2013; Conraths and Deplazes, 2015; Raoul et al., 2015; Knapp et al., 2016; Eckert and Thompson, 2017). Humans play the role of an aberrant IH for *E. multilocularis* and can be infected when ingesting eggs released into vegetables and food by adult worms. Then, the parasite larvae travel to internal organs, mainly the liver (Torgerson et al., 2010; Conraths et al., 2017).

Mitochondrial DNA (mtDNA) has an important role in the taxonomy of *Echinococcus* species. According to mtDNAs, *E. granulosus* species include 10 genotypes (G1-G10) and genetic variation in mtDNAs has also been found within *E. multilocularis* (Bowles et al., 1992; Nakao et al., 2007; McManus, 2013). From early studies, *E. multilocularis* has been divided into two geographical genotypes, M1 (Europe) and M2 (China, Alaska and North America), based on four nucleotide substitutions in the *nad*1 gene (Bowles and McManus, 1993; Okamoto et al., 1995). Nakao and his co-workers found 17 regional haplotypes based on three mt protein-coding genes: cytochrome c oxidase subunit 1 (*cox*1), cytochrome b (*cyt*b), and NADH dehydrogenase subunit 2 (*nad*2) (Nakao et al., 2009). All of those studies show a relatively low mt nucleotide diversity within *E. multilocularis*. To date, more genes have been used for investigating genetic variation in *E. multilocularis*. Although the nuclear genome and the microsatellite targets are also used for molecular markers (Knapp et al., 2008; Umhang et al., 2014; Laurimaa et al., 2015; Karamon et al., 2017), mt genes are more commonly used in the analysis of genetic variation in *E. multilocularis*, because they have a great copy number and evolve at a high rate (Brown et al., 1979; Ciesielski et al., 2016).

In our study, we chose mtDNAs as molecular markers to assess the diversity of *E. multilocularis* in plateau voles (*Neodon/ Microtus fuscus*) and plateau pikas (*Ochotona curzoniae*). In addition to the common mt *cox*1 and *nad*1 genes, NADH dehydrogenase subunit 5 (*nad*5) and adenosine triphosphate subunit 6 (*atp*6) were also included for genetic variation analysis, which provide basic information on molecular epidemiology, genetic variations or differences in *E. multilocularis* in China.

## Materials and methods

### Ethics statement

All animals were handled in strict accordance with good animal practice according to the Animal Ethics Procedures and Guidelines of the People's Republic of China, and the study was approved by the Animal Ethics Committee of Lanzhou Veterinary Research Institute, Chinese Academy of Agricultural Sciences (No. LVRIAEC2012-007). In addition, all mice were handled in strict accordance with the animal protection laws of the People's Republic of China (A Draft of an Animal Protection Law in China released on September 18, 2009).

### Biosecurity statement

The biohazards, biological select agents, toxins, restricted materials or reagents involved in the research have been carried out according to the “Measures for the prevention and control of environmental pollution of hazardous chemicals” issued by the state environmental protection administration (No. 27_2005-03-30) and the “Regulations on the management of medical waste” promulgated by the state council of the People's Republic of China (2003-06-16).

### Sample collection

The specimens of *E. multilocularis* were collected from Jiuzhi County, Golog Autonomous Prefecture, Qinghai Province (33.32° N, 100.53° E) at an average elevation of 4,100 m above sea level. All samples were collected at random in the field amid a “Rodent control program” that was carried out by the local Center for Animal Disease Control and Prevention. Cystic lesions were collected from two intermediate host species: plateau voles and pikas. They were isolated and placed in 70% (v/v) ethanol before being sent to the laboratory at Lanzhou Veterinary Research Institute (LRVI). The lesions were used for DNA extraction, PCR amplification and sequencing to confirm the parasite species and analyze genetic variations. Additionally, one *E. multilocularis* metacestode sample from the Xinjiang Uygur Autonomous Region was preserved by our laboratory.

### DNA extraction

The cystic lesions were rinsed repeatedly using phosphate buffered saline (PBS) to remove the ethanol, and then, the lesions were disrupted using TissueLysers before DNA extraction. With the use of the TIANamp Genomic DNA Kit (TianGen Biotech, Beijing, China), the total DNA was extracted according to the manufacturer's protocols.

### PCR amplification and sequencing of MT genes

Four pairs of primers were designed for *nad*5, *atp*6, *nad*1, and *cox*1 genes (Table 1). A PCR reaction mix included 25 μl Premix Ex *Taq* version 2.0 (Takara Biomedicals, Shiga, Japan), 1 μl DNA template, 1 μl primer F (25 μM), 1 μl primer R (25 μM), and 22 μl ddH_2_O. The PCR was carried out using a standard 3-step cycle: 94°C, 4 min; 35 cycles of 94°C, 30 s; 50–57°C, 30 s' 72°C, 1–2 min; 72°C, 10 min. Each PCR product purified for sequencing was gel-cut and DNA was recovered through a column (AxyPrep DNA Gel Extraction Kit by AxyGen). The purified products were sent to GENEWIZ, Inc. (Beijing, China) to be sequenced using Sanger dideoxy chain termination in an ABI3730 DNA Analyzer.

**Table 1:** Primers for PCR amplification for mt genes of *E. multilocularis* with positions based on a reference sequence (AB018440 in GenBank).

**Gene**	**Primer name**	**Primer sequence (5^′^-3^′^)**	**Primer position**	**Size (bp)**
*nad*5	Emnad5-f	CTATTATGGTGTTAGTTGTTGAC	490–512	1,914
	Emnad5-r	AACCACAGACATATCTATATCG	2382–2403
*atp*6	Ematp6-f	AAGGTGATTAGTTGTCCGT	5613–5731	808
	Ematp6-r	TGCTAACCTACACAACTCC	6502–6520
*nad*1	Emnad1-f	GAGTTTGCGTCTCGATGATAGG	7386–7407	1,126
	Emnad1-r	TCCCCA AAACCCACATTCTAC	8491–8511
*cox*1	Emcox1-f	AGGTTTGACTTTCTCTTTGGTT	9072–9093	1,801
	Emcox1-r	GGCAAATAAACCTAAACAACC	10852–10872

### Sequence alignment and analysis

The open reading frames of all raw nucleotide sequences of the *nad*5, *atp*6, *nad*1, and *cox*1genes of each isolate were assembled, edited and concatenated into a total sequence using the software package SeqMan (DNAStar, Inc., Madison, WI, www.dnastar.com/t-megalign.aspx) and Clustal W2 (online software, http://www.ebi.ac.uk/Tools/msa/clustalw2/, serviced by EBI, the European Bioformatics Institute). The total sequences (one of the sequences having 100% similarity with the others was chosen) were aligned using BioEdit v7.2.3 and ClustalX 1.83 (Thompson et al., 1997; Hall, 1999). The Megalign software (DNAStar, Inc., Madison, WI) was used to calculate the genetic divergence between different haplotypes. In phylogenetic reconstruction, *E. shiquicus* (GenBank accession no. AB159136) was used as an out group, because it was considered the sister species of *E. multilocularis* (Nakao et al., 2007). Bayesian analysis was performed to combine four datasets by using MrBayes 3.1.2 with a general time reversible (GTR) model of DNA substitution and a gamma distribution rate variation across sites (Ronquist and Huelsenbeck, 2003). In this model, a Metropolis-coupled Markov chain Monte Carlo analysis was run for 1 million generations, and trees were sampled every 100 generations. The first one-fourth of 10,000 trees were treated as burn-in. The command was executed until the average standard deviation of the split frequencies was lower than 0.01 (Nakao et al., 2009; Zhao et al., 2013).

Phylogenetic reconstruction was completed using PAUP (phylogenetic analysis using parsimony) version 4.0b10 (Cummings, 2014) by the neighbor-joining (NJ) and maximum parsimony (MP) methods. The NJ tree was performed from HKY85 distances with inverse-squared weighting (power = 2). The MP tree was executed using a heuristic search with TBR branch swapping options and 1,000 random sequence additions. The clade of trees was estimated with 1,000 replicates of bootstrap (BT) analysis. The descriptive tree statistics, tree length (TL), consistency index (CI), retention index (RI), rescaled consistency index (RC), and homoplasy index (HI) for Maximum Parsimonious Tree were engendered (Zhao et al., 2013).

Then, we used MEGA6 to construct a phylogenetic tree based on the *cox*1 gene by maximum likelihood approaches (ML) (GTR+G+I model; 1,000 bootstraps) with the sequences (GenBank accession no. AB477010-AB477012, AB461412- AB461420, AB688125-AB688135, AB777915-AB777921, AB813186-AB813188, KT001423, KT001424, and KY205677-KY205691) downloaded from NCBI (Tamura et al., 2013). The network of *E. multilocularis* based on mt concatenated sequences was calculated using TCS 1.21 software (Clement et al., 2000).

## Results

### *E. multilocularis* cysts

A total of 102 voles (*N. fuscus*) and 49 pikas (*O. curzoniae*) were captured in Jiuzhi County. Of these animals, 32 (30 voles, infection rate: 29.41%; 2 pikas, infection rate: 4.08%) had cystic lesions in their viscera. From these 32 individuals, 39 cysts were isolated. Twenty-seven cysts were found only in the liver, and the others were found not only in the liver but also in the lungs, the intestinal wall and the muscles (Table 2).

**Table 2:** Haplotypes of *E. multilocularis* isolates, their species, and their organ location in the 32 samples.

**Samples**	**Host and Cyst location**					**Haplotype^a^**
	**Host**	**Liver**	**Lung**	**Intestinal wall**	**Muscle**
Cyst1	Vole1	+	–	–	–	JZ01
Cyst2	Vole2	+	–	–	–	JZ02
Cyst3	Vole3	+	–	–	–	JZ03
Cyst4	Vole4	+	–	–	–	JZ01
Cyst5	Vole5	+	–	–	–	JZ04
Cyst6	Vole6	+	–	–	–	JZ05
Cyst7	Vole7	+	–	–	–	JZ05
Cyst8	Vole8	+	–	–	–	JZ04
Cyst9	Vole9	+	–	–	–	JZ06
Cyst10	Vole10	+	–	–	–	JZ03
Cyst11	Vole11	+	–	–	–	JZ07
Cyst12	Vole12	+	–	–	–	JZ04
Cyst13	Vole13	+	–	–	–	JZ05
Cyst14	Vole14	+	–	–	–	JZ01
Cyst15	Vole15	+	–	–	–	JZ04
Cyst16	Vole16	+	–	–	–	JZ03
Cyst17	Vole17	+	–	–	–	JZ03
Cyst18	Vole18	+	–	–	–	JZ01
Cyst19	Vole19	+	–	–	–	JZ05
Cyst20	Vole19	–	+	–	–	JZ05
Cyst21	Vole20	+	–	–	–	JZ04
Cyst22	Vole21	+	–	–	–	JZ01
Cyst23	Vole22	+	–	–	–	JZ03
Cyst24	Vole23	+	–	–	–	JZ05
Cyst25	Vole24	+	–	–	–	JZ08
Cyst26	Vole25	+	–	–	–	JZ05
Cyst27	Vole26	+	–	–	–	JZ01
Cyst28	Vole27	+	–	–	–	JZ12
Cyst29	Vole27	–	+	–	–	JZ12
Cyst30	Vole27	–	–	+	–	JZ12
Cyst31	Vole27	–	–	–	+	JZ12
Cyst32	Vole28	+	–	–	–	JZ09
Cyst33	Vole28	–	+	–	–	JZ10
Cyst34	Vole29	+	–	–	–	JZ03
Cyst35	Vole30	+	–	–	–	JZ13
Cyst36	Vole30	–	+	–	–	JZ13
Cyst37	Pika1	+	–	–	–	JZ11
Cyst38	Pika1	+	–	–	–	JZ11
Cyst39	Pika2	–	+	–	–	JZ11

a *Based on complete concatenated nad5, atp6, nad1 and cox1 nucleotide sequences*.

### Characterization of haplotypes and phylogenetic trees analysis

The combined sequences of *nad*5 (1,575 bp), *atp*6 (516 bp), *nad*1 (894 bp) and *cox*1 (1,608 bp) genes were distributed into 15 haplotypes including one from Xinjiang Uygur Autonomous Region and one from Yushu Tibetan Autonomous Prefecture (Table 2), which were designated as JZ01 to JZ13 (haplotypes from Jiuzhi), XJ (haplotype from Xinjiang), and YS (haplotype from Yushu), respectively. When coming from a single gene, the number of haplotypes decreasedto 7 in *nad*5, 2 in *atp*6, 4 in *nad*1, and 11 in *cox*1 (Table 3). Phylogenetic analyse showed that the genetic diversity of individual gene changes was 0.1–0.4% (*nad*5), 0.2–0.4% (*atp*6), 0.1–0.6% (*nad*1), and 0.1–0.4% (*cox*1). Within the four genes, *cox*1 had the most mutation sites (with 9 sites), followed by 7 sites in *nad*5, 2 sites in *atp*6 and 2 sites in *nad*1 (Table 4). When we compared the sequences with a reference sequence for partitioned *nad*5, *atp*6, *nad*1, and *cox*1 genes, there were only two haplotypes in the four genes. The genetic distance for JZ01 wa 99.7, 99.8, 99.9, and 99.8%, and for JZ03, it was 99.7, 99.6, 100, and 99.9%. The sequence analysis with the reference sequence in this study further predicted that all of the samples or specimens isolated from plateau voles and pikas belonged to *E. multilocularis*.

**Table 3:** The number of haplotypes based on the *nad*5, *atp*6, *nad*1, and *cox*1 genes.

**Haplotype**	**Gene**	**GenBank accession number**
EmJZ01	*nad*5	MH259779
EmJZ03	*nad*5	MH259780
EmJZ06	*nad*5	MH259781
EmJZ08	*nad*5	MH259782
EmJZ12	*nad*5	MH259783
EmJZ13	*nad*5	MH259784
EmXJ	*nad*5	MH259785
EmJZ01	*apt*6	MH259786
EmJZ03	*apt*6	MH259787
EmJZ01	*nad*1	MH259775
EmJZ03	*nad*1	MH259776
EmJZ07	*nad*1	MH259777
EmJZ11	*nad*1	MH259778
EmJZ01	*cox*1	MH259764
EmJZ02	*cox*1	MH259765
EmJZ03	*cox*1	MH259766
EmJZ04	*cox*1	MH259767
EmJZ05	*cox*1	MH259768
EmJZ06	*cox*1	MH259769
EmJZ09	*cox*1	MH259770
EmJZ10	*cox*1	MH259771
EmJZ11	*cox*1	MH259772
EmXJ	*cox*1	MH259773
EmYS	*cox*1	MH259774

**Table 4:** Mutation sites in the complete *nad*5, *atp*6, *nad*1 and *cox*1 gene sequences among different *E. multilocularis* (*E.m*.) haplotypes.

**Haplotype**	**Mutation sites**																			
	***nad*****5**							***atp*****6**		***nad*****1**		***cox*****1**								
	**297**	**372**	**567**	**819**	**1363**	**1379**	**1510**	**60**	**358**	**137**	**870**	**46**	**70**	**461**	**514**	**873**	**1107**	**1329**	**1501**	**1596**
*E.m*. ref.	T	T	C	G	T	A	T	T	A	C	A	A	T	C	G	T	C	G	G	T
JZ01	–	C	–	A	C	G	C	G	–	–	G	–	–	–	–	–	–	A	C	G
JZ02	–	C	–	A	C	G	C	G	–	–	G	–	G	T	–	–	–	A	–	–
JZ03	–	C	T	–	C	G	–	G	G	–	–	–	–	–	–	–	T	A	–	–
JZ04	–	C	–	A	C	G	C	G	–	T	–	–	–	T	–	–	–	A	–	–
JZ05	–	C	T	–	C	G	–	G	–	T	–	–	–	–	–	–	–	A	–	–
JZ06	–	C	T	–	C	G	–	G	–	T	–	–	–	–	–	–	–	A	–	–
JZ07	–	C	T	–	C	G	–	G	–	T	G	–	–	–	–	–	T	A	–	–
JZ08	–	C	T	–	C	G	–	G	G	–	–	–	–	–	A	–	–	A	C	–
JZ09	–	C	T	–	C	G	–	G	G	–	–	–	–	–	A	–	T	A	–	–
JZ10	–	C	T	–	C	G	–	G	G	–	–	C	–	–	–	–	T	A	–	–
JZ11	–	C	–	A	C	G	C	G	G	–	–	–	–	–	–	C	T	A	–	–
JZ12	G	C	T	–	C	G	–	G	G	–	–	–	–	–	–	–	T	A	–	–
JZ13	–	C	–	A	C	G	C	G	–	–	G	–	–	–	–	–	–	A	C	G
YS	–	C	–	A	C	G	C	G	–	–	–	–	–	–	–	–	–	–	–	–
XJ	–	–	–	–	–	–	–	G	–	–	–	–	–	T	–	–	–	A	–	–

*“–”Indicates the nucleotide is same as the E. multilocularis reference mtDNA sequence (AB018440).

There were a number of changes in amino acids, with synonymous substitutions in 7 sites, from 460S to 460N (*nad*5, haplotype XJ); from 120R to 120G (atp6, haplotypes JZ03, JZ08-JZ12); from 46A to 46V (nad1, haplotypes JZ04-JZ07); from 16K to 16Q (*cox*1, haplotype JZ10); from 24L to 24V (*cox*1, haplotype JZ02); from 46E to 46Q (*cox*1, haplotype JZ06); from 153A to 153V (*cox*1, haplotypes JZ02, JZ04, XJ); from 172V to 172I (*cox*1, haplotypes JZ09, JZ10); from 443I to 443M (*cox*1, haplotypes YS); from 501A to 501P (*cox*1, haplotypes JZ01, JZ13) and from 532F to 532L (*cox*1, haplotypes JZ01, JZ13). There was a heterogeneous substitution in 504 (*cox*1), from W to R (haplotypes JZ01, JZ02, JZ04, JZ11, JZ13, YS).

**Figure 1:**
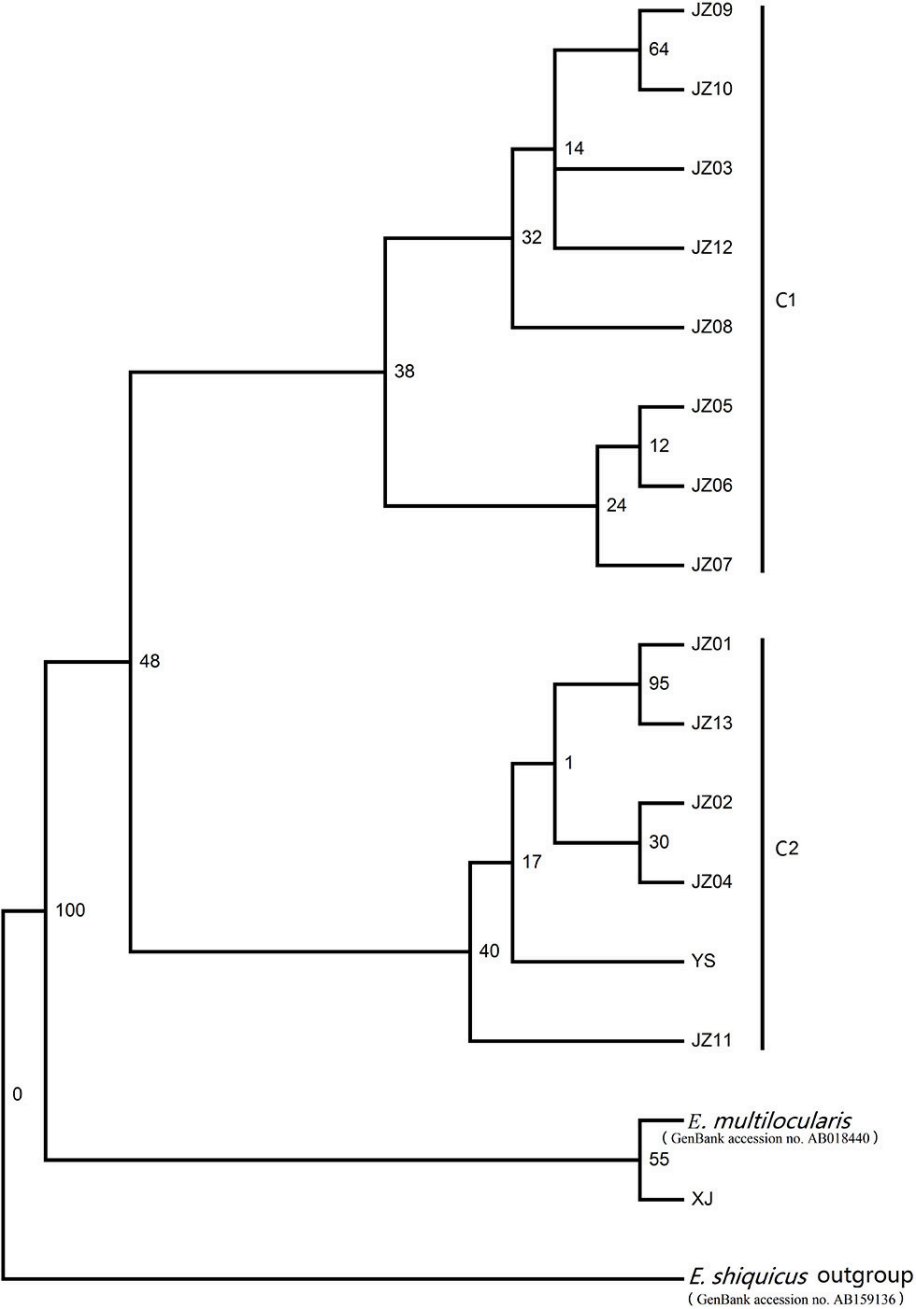
Maximum parsimony (MP) tree constructed with the PAUP version 4.0b10 based on the concatenated nucleotide sequences of the *nad*5, *atp*6, *nad*1, and *cox*1 genes.

**Figure 2:**
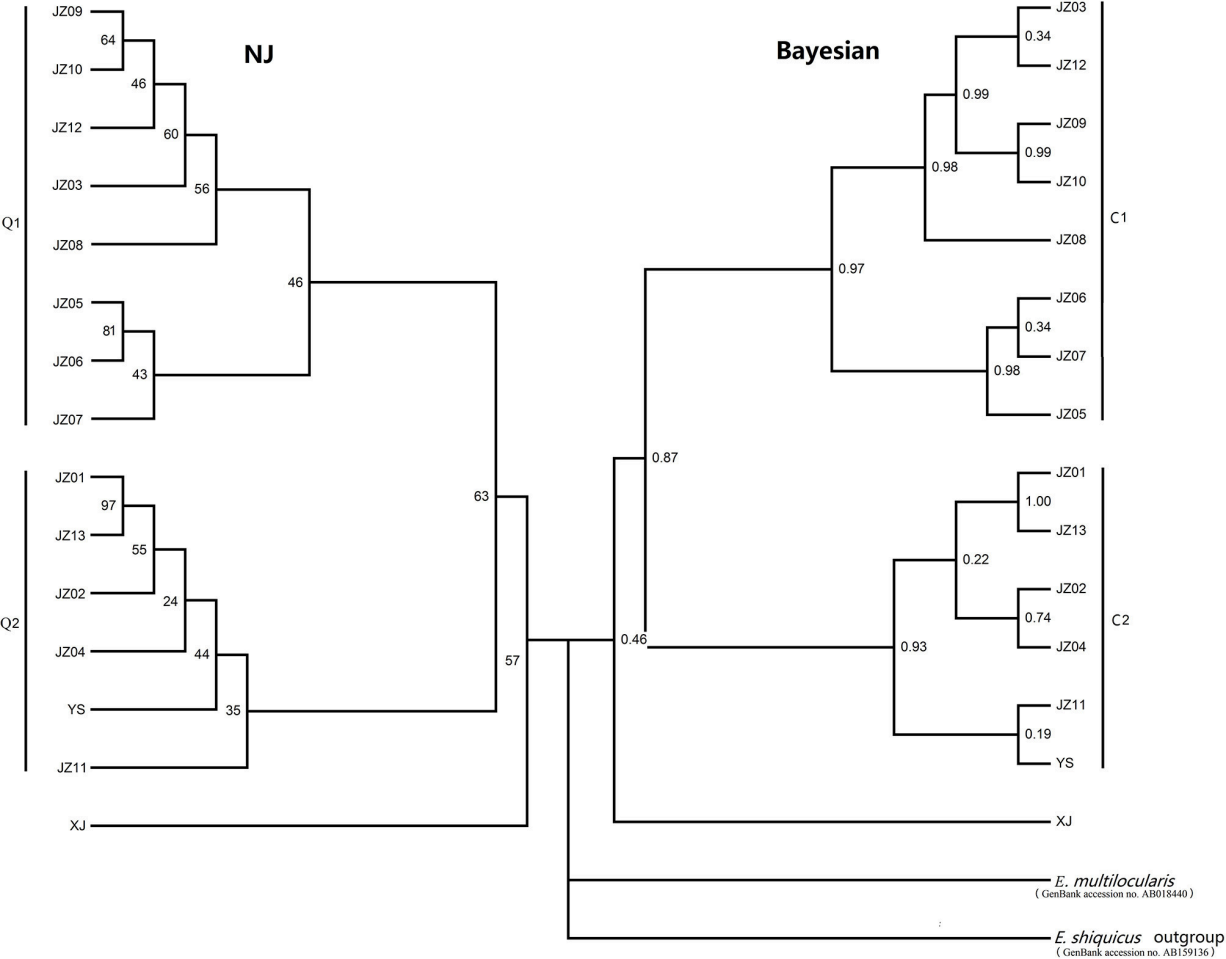
Haplotype cladograms of *E. multilocularis* inferred by the neighbor-joining (NJ) and Bayesian methods using the concatenated nucleotide sequences of the *nad*5, *atp*6, *nad*1, and *cox*1 genes.

**Figure 3:**
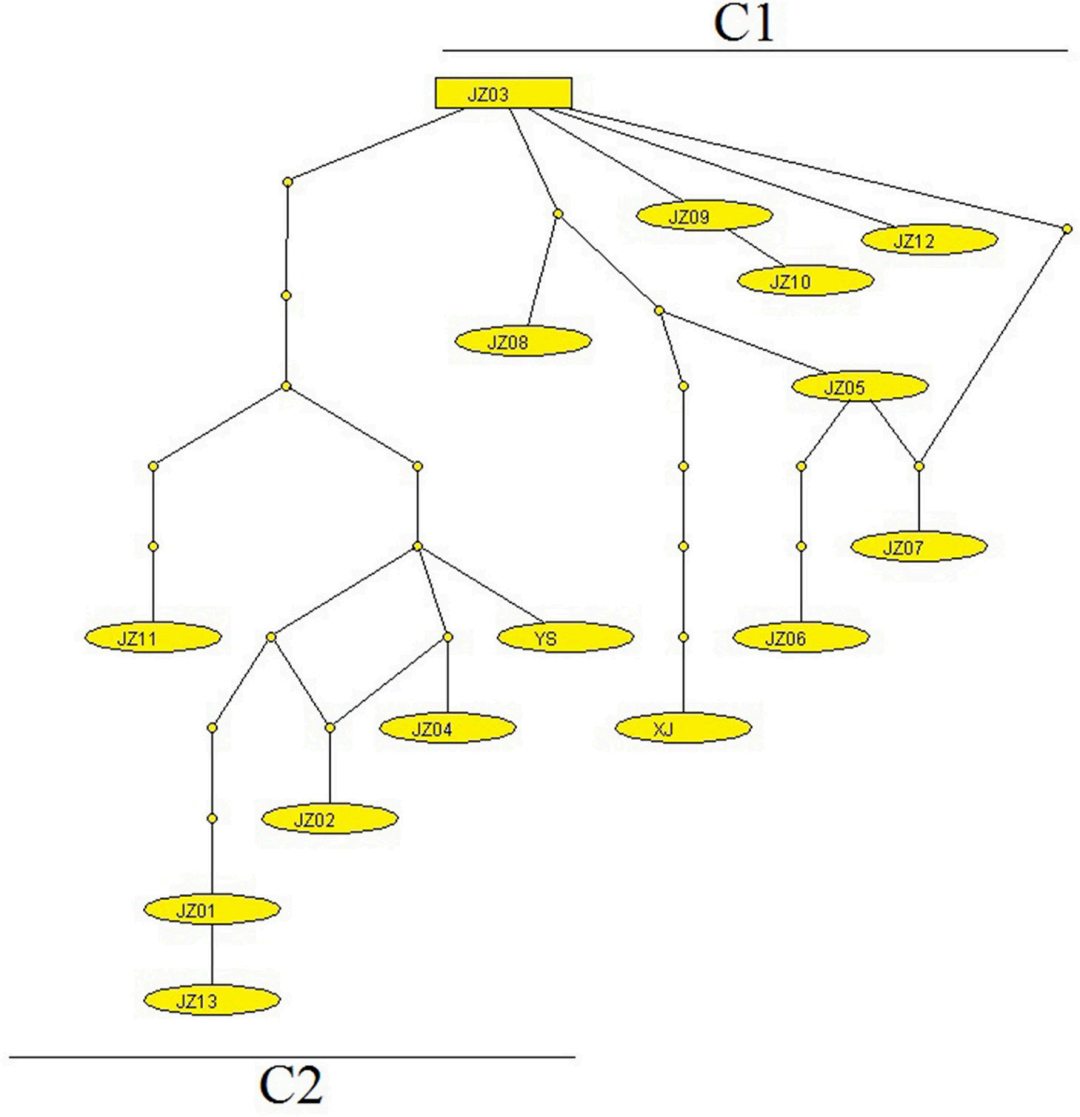
The network of *E. multilocularis* generated using mt concatenated genes collected from voles and pikas. Each link between haplotypes indicates one mutational difference and the unlabeled nodes point out that inferred steps not found in the samples.

The phylogram based on the maximum parsimony method precisely revealed that the 14 haplotypes were divided into two large haplogroups: the JZ03, JZ05-JZ10, and JZ12 haplotypes had a close relationship with each other (classified as C1 haplogroup) and the rest of the haplotypes gathered together (classified as C2 haplogroup) (Figure 1). The haplotype XJ from Xinjiang Province is at the base of the phylogenetic tree. In this phylogenetic tree, the bootstrap value of three nodes was almost the same and was low. The maximum genetic diversity between the XJ haplotype and C1 haplogroup reached 0.2%, and the divergence between haplotype XJ and C2 was 0.2%. When compared haplogroup C1 with C2, the rate of pairwise divergence was the same. Haplotype cladograms of *E. multilocularis* were carried by Bayesian and NJ methods using the combined nucleotide sequences of the *cox*1, *nad*1, *nad*5, and *atp*6 genes. The two methods both showed a similar result with two clades (Figure 2). Furthermore, the network of those haplotypes also divided into the two same groups (Figure 3). Compared to previous studies of the genetic diversity of *E. multilocularis* based on the *cox*1 gene, we found that the sequences could be divided into 4 groups; Mongolia strain, North American strain, European strain and Asian strain. The highest genetic divergence is 1.3–1.7% (samples collected from Inner Mongolia, Mongolia and Russia), followed by 0.6–0.8% (samples collected from the USA: Alaska and Indiana), 0.3–0.6% (samples collected from Austria, Estonia, France, Poland, Slovakia and Russia) and 0.1–0.4% (samples collected from China: Sichuan, Kazakhstan, Japan: Hokkaido). The genetic distance within the same group is 0.1–0.3% (Mongolia strain), 0.4% (North American strain), 0.1–0.3% (European strain), and 0.1–0.4% (Asian strain) (Nakao et al., 2009; Ito et al., 2010; Konyaev et al., 2012; Laurimaa et al., 2015; Karamon et al., 2017). Similarly, according to the phylogenetic tree based on the *cox*1 gene constructed by MEGA6, all haplotypes clustered together with the haplotypes downloaded from NCBI (samples collected from China: Sichuan, Kazakhstan, Japan: Hokkaido), belonging to the Asian clade (not shown).

## Discussion

The genus *Echinococcus* has a unique reproductive cycle, including a sexual reproduction in the adult stage and asexual proliferation in the larval phase. Because of this reproductive model, *Echinococcus* has genetic monomorphism in local populations (Lymbery and Thompson, 1996; Haag et al., 1999; Nakao et al., 2007). In early studies, *E. multilocularis* was recognized as a subspecies of *E. granulosus*, but Rausch and Nelson thought that *E. multilocularis* had unusual morphological and biological peculiarities and should be a separate genus. Then, phylogenetic analyses based on the mt genes also indicated that *E. multilocularis* was different compared with *E. granulosus* (Lukashenko and Zorikhina, 1961; Rausch and Nelson, 1963; Tappe et al., 2010). Now the genus *Echinococcus* includes at least 10 species: *E. granulosus, E. equinus, E. ortleppi, E. Canadensis, E. intermedius, E. felidis, E. multilocularis, E. vogeli, E. oligarthr*a, and *E. shiquicus* (Thompson, 2008, 2017; Nakao et al., 2013; Thompson and Jenkins, 2014).

Until now, a majority of published data on the gene diversity and phylogenetic analysis of the genus *Echinococcus* have been based on short mt genes, especially the *cox*1 and *nad*1 genes. From early studies, nine genotypes were classified in *E. granulosus s. l*. based on the diversity of the mt genes *cox*1 and *nad*1, and these two genes were applied in *E. multilocularis* genetic variation analysis (Bowles et al., 1995; Tappe et al., 2010; Romig et al., 2017). In our study, we used the 4 full-length sequences of mt genes to analyze the diversity of *E. multilocularis* on the local scale. Herein, the sequence divergence within *E. multilocularis* wase 0.1–0.4% for *nad*5, 0.2–0.4% for *atp*6, 0.1–0.6% for *nad*1, and 0.1–0.4% for *cox*1. The result showed that the *nad*1 gene had more diversity than those of the other genes, which is in contrast with previous studies showing that *atp*6 had a comparatively more diversity within the *Echinococcus* spp. and *E. multilocularis* (Yang et al., 2005; Nakao et al., 2007). However, our results were in keeping with a previous study based on the *cox*1 gene. The variation in the *nad*5 gene showed a consistent results with the published data of the other parasites (Zhao et al., 2014; Lou et al., 2015). Regarding amino acid, *cox*1 exhibited the highest frequency of substitution (0.7476%, 4 substitution sites/535 sites), followed by *apt*6 (0.5814%, 1/172), *nad*5 (0.3817%, 2/524), and *nad*1 (0.3367%, 1/297). According to the results, even though the analysis of the *cox*1 gene was more meaningful from an evolutionary standpoint, the phylogenetic tree based on only one gene was not accurate. The phylogenetic trees analysis revealed that all 15 haplotypes divided into 3 groups. The 14 samples isolated from Qinghai-Tibet plateau divided into two clades, and the haplotype isolated from Xinjiang province was in a separate group. In C1, the JZ09 haplotype was isolated from the lungs of voles, the JZ12 haplotype was isolated from the intestines of voles, and the remaining haplotypes were found in the liver of voles. In C2, the JZ13 haplotype was isolated from the lungs in voles, the JZ11 haplotype was isolated from the lungs of pikas, and the others were found in the liver of voles. The phylogram indicted that the different intermediate hosts and parasitic sites had no effect on the genetic diversity of *E. multilocularis* on a local scale. From the results of the genetic divergence within haplotypes based on the *cox*1 gene, we observed that the European strain has the lowest genetic diversity, and the Asian strain and Mongolia strain have the highest diversity. This also implied that the relationship maybe not important between geographical distances and genetic distances.

In conclusion, our study, based on the mitochondrial complete coding genes *nad*5, *atp*6, *nad*1, and *cox*1, shows a relatively low genetic variation among the samples from voles and pikas on a local scale. The genetic variation is higher in *nad*1 than that in the other three mt genes, and the concatenated mt sequences of the *cox*1, *nad*1, *nad*5, and *atp*6 genes are useful in phylogenetic reconstruction within *E. multilocularis* isolates.

## Author contributions

JL, LL, HY, and WJ conceived and designed the experiments. JL, LL, and YF performed the experiments and the data analyses. JL prepared the figures and wrote the manuscript, WJ and HY provided improving paragraphs and BF and XZ provided very constructive suggestions for revisions.

### Conflict of interest statement

The authors declare that the research was conducted in the absence of any commercial or financial relationships that could be construed as a potential conflict of interest. The handling editor declared a past co-authorship with one of the authors DB.
